# The role of community-based volunteers in integrating interactive playful parenting interventions for early childhood development in rural community systems for health in Zambia: a qualitative study

**DOI:** 10.1186/s12875-025-03119-y

**Published:** 2025-12-02

**Authors:** Malizgani Paul Chavula, Chitalu Miriam Chama-Chiliba, Mwimba Chewe, Zewelanji Serpell, Patricia Maritim, Peter Hangoma

**Affiliations:** 1 Department of Community and Family Medicine, School of Public Health, University of Zambia, Lusaka, Zambia; 2 Department of Epidemiology and Global Health, Umeå University, Umeå, SE Sweden; 3 Institute of Economic and Social Research, University of Zambia , Lusaka, Zambia; 4 Department of Health Policy and Management, School of Public Health, University of Zambia, Lusaka, Zambia; 5 UNICEF Zambia, UN House, Alick Nkhata Road, P.O. Box 33610, Lusaka, Zambia; 6 Chr. Michelsen Institute (CMI), Bergen, Norway

**Keywords:** Collaboration, Early child development, Integration, Early childhood development, Rural community system for health

## Abstract

**Background:**

Early childhood development (ECD) programmes play a crucial role in child development through the promotion of health, nutrition, early education, and playful parenting interventions. Community-based volunteers (CBVs) also play an essential role in facilitating the integration of ECD into rural community systems for health to promote interactive playful parenting for early childhood development. However, little is known about the role of CBVs integrating interactive playful parenting interventions for early childhood development and health in Zambia’s community systems for health. This study explores the role of community-based volunteers in integrating interactive playful parenting interventions for early childhood development in rural community systems for health in Zambia.

**Methods:**

This was a qualitative study conducted in Katete and Petauke districts in Eastern Province, Zambia. We conducted 38 qualitative interviews including 12 focus group discussions (FGDs) with parents/caregivers and CBVs, 18 key informant interviews (KIIs) with district government and NGO stakeholders involved in the implementation, and 8 in-depth interviews (IDIs) with representatives from the Neighbourhood Health Committee (NHC). Data was analysed using thematic analysis guided by five domains of Atun’s conceptual framework on the integration of innovations into health systems: nature of the problem, attributes of the intervention, adoption system, broad system characteristics, and broad context.

**Findings:**

For the nature of the problem, the stakeholders recognized ECD as a cross-cutting issue and highlighted the need for the delivery of ECD interventions that foster playful parenting while emphasizing the need for trained manpower to deliver these interventions. The attributes of the ECD interventions included the design of an ECD training package, provision of capacity-building training and the distribution of training materials and resources to support implementation. The degree of adoption of ECD interventions into community systems for health depended on actors’ perspectives, including community leadership, parents, and teachers on ECD activities. The broader system characteristics shaping ECD integration included district-based multisectoral collaboration in delivery of ECD, community-based collaboration, education and linkages on ECDs. Additionally, the availability of community structures played a significant role in shaping the delivery of ECD activities, while school administration was instrumental in coordinating ECD programmes. Healthcare workers played a key role in providing supportive supervision for delivery of ECD interventions. The broader context influencing the integration of ECD interventions was shaped by the policy and legal environment, political commitment to deliver ECD interventions, challenges related to inadequate allocation of human, financial, and material resources. Furthermore, the lack of infrastructure development posed a barrier to collaborative efforts in delivering ECD services effectively.

**Conclusion:**

The integration of playful and interactive parenting highlights both barriers and facilitators, particularly regarding resources, personnel, and infrastructure. Addressing these challenges requires collaborative and coordinated efforts among the health, education, and social sectors to enhance the integration of ECD interventions. Strengthening ECD service delivery further necessitates increased resource allocation, expanded capacity building initiatives, and infrastructure development to support holistic child development and health.

**Supplementary Information:**

The online version contains supplementary material available at 10.1186/s12875-025-03119-y.

## Introduction

Globally, early childhood development (ECD) interventions have been recognised as a pillar in laying a strong foundation for children’s academic performance, social development, economic productivity, and overall contribution to society [1]. Several studies have shown that the formative years, particularly the first three years of life, are critical for brain development and function, with long-term effects that are shaped by environmental conditions [1, 2]. The global health agenda, including the Sustainable Development Goals (SDGs), stress the need for countries to implement and achieve ECD targets for children under the age of five to improve human capacity development [1].

In low middle-income countries (LMICs), CBVs are involved in the delivery of vertical ECD programmes to promote the healthy development of children through nutrition support, childhood services such as vaccinations, early childhood education, and counselling services [3]. However, recent evidence suggests that parenting interventions, which directly enhance early child learning and strengthen parent–child interactive relationships, are more effective in improving early cognitive, language, motor, emotional, and social development [4, 5]. Parents and guardians are the primary caregivers for young children, ands studies have shown that interactive parent–child relationships and support for learning during the earliest years of life are crucial for promoting early childhood development [5]. Promoting early child development requires a multidimensional approach that integrates health, nutrition, care, early learning, and social protection [1]. However, no single sector incorporates all these dimensions, and existing interventions remain fragmented, and lack collective ownership and advocacy [1].

Community health system refers “the set of local actors, relationships, and processes engaged in producing, advocating for, and supporting health in communities and households outside of, but existing in relationship to, formal health structures” [6]. Despite the essential role of CBVs in promoting ECD, limited integration of ECD programmes within the rural health systems continues to pose a significant challenge. Due to the shortage of healthcare providers who can comprehensively deliver ECD services to communities, community-based volunteers (CBVs) from different sectors including health (CHWs), education (volunteer teachers), community development, and agriculture become key actors in enhancing integration of ECD services into rural communities. CBVs serve as an additional workforce, helpful in rendering services to underserved populations, thereby strengthening the health system’s capacity to address financial and human resource shortages in resource-constrained settings [7].

CBVs play a critical role in the integration of ECD interventions within rural or local health systems through home visits and service provision in other communal settings [1]. They are trained to provide information to parents aimed at encouraging them to prioritise child development, including physical health, school performance, emotional regulation and overall wellbeing. Studies in LMICs confirm that CBVs continue to play a critical role in providing child services [8]. They have been instrumental in creating positive behavioural change towards the uptake of child health, prevention and treatment, social, and nutrition interventions [9]. CBVs tend to enjoy support from various community actors, and this provides legitimacy for the services they provide [8]. Further, these pre-existing community-level support and legitimacy systems allow CBVs and the child health interventions they are delivering to be positively received by community members. Some of the roles played by CBVs include identifying potential beneficiaries of child health services, following up on expectant mothers and women who have delivered, ensuring appropriate nutrition and care services are provided, provision of counselling, and general demand creation for these critical services [10].

Despite recognising the positive contribution of the CBVs in delivering various forms of child services, they continue to face numerous challenges in executing their duties. Some of these obstacles have been widely documented, including unclear reporting systems, inadequate training, poor supervision, lack of job aids, lack of incentives, and difficulties adapting to new contexts, to mention but a few [11]. Furthermore, their actual workload is unclear as it has also been reported that CBVs tend to be the same people operating under different names depending on the programme and related Ministry [7]. The CBVs move from one programme to another in search of opportunities, and this ultimately affects programme performance. The CBVs’ challenges are linked to the broader policy environment as well as the local contextual community factors that require attention to ensure successful delivery and acceptability of interventions to enhance child services at the community level [12]. Therefore, it is important to consider the challenges that CBVs face, including their workload, available resources, and existing gaps between what they have and what they need to ensure the successful delivery of a community-based ECD parenting support programme.

### Community-based volunteers’ landscape in Zambia

Zambia, just like other LMICs, has an active and diverse cadre of CBVs across various sectors including health, education, community development and agriculture etc. The term CBV in the context of this study, refers to local individuals that deliver services within their communities in the areas of community development, mother, and child health and/or social welfare without receiving any remuneration for these services (MoH, 2014). CBVs perform numerous functions and have been recognised as key players in national development efforts. Some of the CBVs involved in providing health services for children are located at the Ministry of Health (MOH), such as the Community Health Workers (CHWs), Community Health Assistants (CHAs), Safe Motherhood Action Groups (SMAGs) and Growth Monitor Promoters (GMPs). The Ministry of Community Development and Social Services (MCDSS) have Community Welfare Assistant Committees (CWACS), whilst the Ministry of Education (MOE) has volunteer teachers. These volunteers perform similar tasks pertaining to child services, but there are widely documented challenges. The CHAs are formerly trained for 18 months and employed by government to manage community health programmes [12]. However, the majority of CBVs are not adequately remunerated for their work and where funding is available It is partner dependent, not standardised and inconsistent.

There is a considerable overlap in service provision and delivery as most CBVs are associated with various programmes across different ministries [13]. For instance, SMAGs are associated with the maternal and child health programme, whereas GMPs are associated with nutrition programmes. Community Welfare Assistant Committees, on the other hand, mainly operate on the Social Cash Transfer programme and the Public Welfare Assistance Scheme. Despite this overlap in service delivery, there is no formal integration of service delivery or referral systems across the different programmes, which may pose a challenge for CBVs that work on multiple programmes [13].

Further, some CBVs are not fully integrated into the systems that they supposedly represent [8]. There are also accounts of CBVs facing resistance from other workers; discrimination of CBVs based on social, gender, and economic status; ineffective incentive structures; inadequate infrastructure and supplies; and hierarchical and parallel communication structures. Additionally, there is also no legal framework to guide and standardise the engagement of this important human resource subsector [12]. There are no clear guidelines on the CBV’s roles, tasks, and responsibilities regarding child services that they should provide. Efforts to provide coordinated policy response to CBV challenges have often been fragmented and have not yielded the intended results over the years.

### Early childhood development (ECD): interactive playful parenting programme

In December 2019, the Ministry of Health in collaboration with UNICEF and other local implementing partners drawn from various sectors including education, social welfare and community development, began the implementation of Early Childhood Development (ECD) Playful Parenting Programme in Eastern Province, Zambia. It was a joint initiative arising from a shared recognition of the importance of early childhood care and stimulation on the holistic development of young children [14]. The early childhood development playful parenting programme for parents and their infants aged 0–3 years was piloted in two selected districts (Katete and Petauke) in Zambia. It sought to improve playful interactions by targeting 50,000 caregivers through proven community-based approaches that involved the rollout of behaviour change communication messages that are targeted towards parents and caregivers. This programme leverages the comparative advantage of CBVs and ensures that they are the primary cadre delivering multisectoral ECD activities. CBVs also provide counselling sessions at home and during community meetings in the Insaka (Community Hall). The community halls (Insaka) provide a platform for conducting community sensitisation and practical sessions on nutrition programmes. The intervention was multisectoral in nature, where the Ministry of Education, through the preschool and volunteer teachers were providing ECD activities. These activities included play, delivery of elementary education, and provision of food starting at the age of 3 years.

A formative study was conducted to understand and map CBVs working in different sectors in relation to their roles, facilitators, and barriers that influence their service delivery in rural health settings [14]. This study also provides a clear indication of the status of key indicators for the community-based integrated ECD programme. The results highlight significant scope for improvement in areas such as nutrition, health, child protection, responsive caregiving, early stimulation, and parent counselling outcomes and outputs [14]. However, very little evidence exist regarding factors shaping the integration of playful parenting intervention to promote early childhood development by three years of life in community systems for health in Zambia. Therefore, this study sought to explore the conditions influencing the integration of interactive playful parenting for early childhood development and health in community systems for health in Zambia in the context of the Pilot LEGO Playful Parenting Programme in Zambia.

**Fig. 1:**
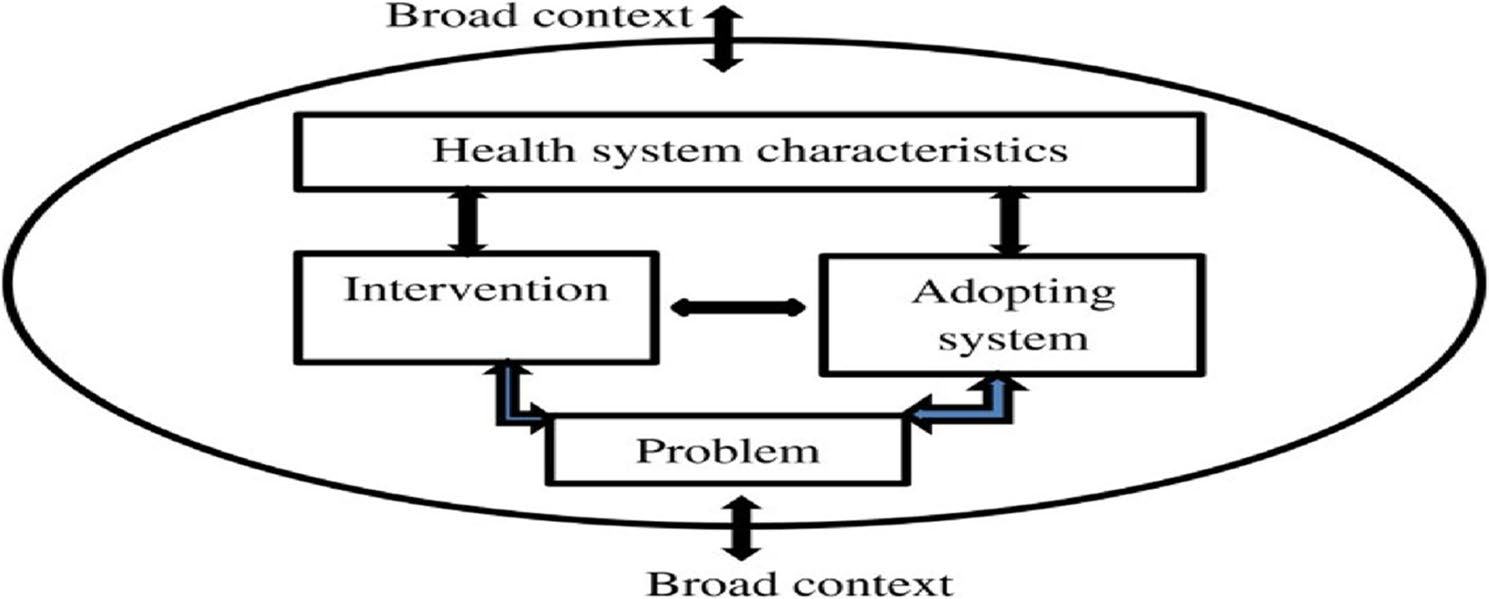
Conceptual Framework for Analysing the Integration of ECD [15]

The study adopts Atun’s [15] framework for integrating innovation into the health system, as it provides a structured approach to analyse complex interventions such as ECD interventions. According to this framework, the integration and implementation of new health interventions are influenced by the actor’s perceptions of the nature of the problem being addressed, the intervention itself, the system into which the intervention is being adopted, health system characteristics, and the broad context (Fig. 1). We define integration as the process through CBVs implemented playful interventions to promote ECD in rural community systems for health as part of their normal daily duties.

In the context of ECD, the nature of the problem includes deficiency and demand for ECD interventions, and shortage of human resources to deliver ECD interventions. Attributes of the intervention include ECD training package, training process perspectives, and distribution of ECD training materials, and resources, as well conducting education and counselling in communities and during homes visits. Integration is influenced by the level of adoption systems that includes the perspectives of key players (policy implementer, teachers, parents, or community) and the programme’s compatibility with broad system characteristics such as district-based multisectoral collaboration in delivery of ECD programme. Others are community-based collaboration, education, and linkages on ECDs, community-based social accountability, and coordination on delivery of ECD, school administration led-supervision and collaboration, health facility led-supportive supervision, challenges, and opportunities, inadequate monitoring, and evaluation systems and the broad context which includes, availability of policies, laws, resources political commitment, and infrastructure development. This framework has been applied in similar contexts, including studies on the implementation of comprehensive sexuality education (CSE) in education [16], and integrating national community-based health worker programmes into health systems in LMICs [12].

## Methodology

### Study setting and design

The Government of the Republic of Zambia, with support from UNICEF implemented the Insaka integrated ECD programme through interactive parenting in the Katete and Petauke districts of Zambia [14]. The programmes aim to strengthen the capacity of parents and caregivers in Zambia to engage in nurturing care practices and playful parenting. The Insaka model focuses on parents and caregivers, strengthening their skills in using play activities to help children develop cognitive, physical, communication, and social/emotional skills. Through the programme, CBVs are trained to support ECD services, including nurturing care, play, and counselling techniques conducted in homes and communities [14]. The study was conducted in Katete and Petauke districts located in the Eastern Province of Zambia. These districts were selected because they served as pilot sites for integrating playful parenting into ECD programmes to address existing service gaps. This context made them ideal for exploring community-based approaches in the delivery of ECD programmes. These sites are rural settings, and with limited access to formal ECD services. The programme’s focus on playful parenting and nurturing care aligns with local efforts to strengthen caregiver capacity through CBVs trained across health, education, and community development sectors.

The study population included CBVs and other stakeholders such as district ECD implementers, teachers, healthcare providers, and community leaders who were involved in the programme. This study adopted a qualitative case study approach to explore the role of community-based volunteers in integrating interactive playful parenting interventions for early childhood development in rural community systems for health in Zambia. The case study approach allows for an in-depth investigation of contemporary phenomena within a real-life context, where the boundaries between phenomena, and setting are not clear and when multiple sources of evidence are used [17]. The case study design was considered appropriate because ECD interventions are complex and are shaped by contextual issues within local health systems. This design facilitated an analysis of the relationships among actors involved in implementing ECD within community settings.

### Data collection methods, and participant selection strategy

A total of 38 qualitative interviews were conducted, including 12 focus group discussions (FGDs), 18 key informant interviews (KIIs), and 8 in-depth interviews (IDIs). Participants included district coordinators, and community-based actors involved in ECD and health activities. The district-based implementers included selected government departments such as social welfare, community development, and health, as well as representatives from non-governmental organisations (NGOs) representatives. The district level actors are involved in coordinating the multisectoral delivery of ECD and health activities. Additional interviews were conducted with teachers, healthcare providers, CBVs, neighbourhood health committee leaders, and parents (see Table 1). All these stakeholders played a crucial role in the multisectoral implementation and support of ECD interventions. Data collection took place between April and June 2021 in Katete and Petauke districts, approximately 18 months after the commencement of the implementation of ECD activities. Participants were purposively selected based on their active involvement in the implementation of the pilot ECD and health activities for at least 18 months. We used semi-structured data collection tools to facilitate discussions, allowing for a flexible yet structured exploration of key issues. The first author, supported by trained research assistants, conducted all interviews. The research team had experience in collecting qualitative data.

**Table 1:** Participant information

Types of Interviews		Role/relevance	Total
KIIs	District programme implementers	Involved in the coordination of the ECD programme	10
KIIs	teachers	Supervision of volunteer teachers in the delivery of ECD activities	4
KIIs	Healthcare providers	Involved in the delivery of ECD, and training and supervision of CHWs engaged in ECD activities	4
IDIs	Ward development committee leaders	Serve as representatives of the community, providing local leadership and ensuring social accountability in social development	4
IDIs	NHCs leaders	Supervise and monitor CBVs	4
FGDs	CBVs	lead ECD sensitization efforts, refer cases to the clinic, and act as the link between the community and the clinic	8
FGDS	Caregivers (parents)	ore beneficiaries of the ECED intervention on behalf of children	4
Total Interviews			38

### Data analysis

All audio-recorded interviews were transcribed verbatim. The lead author (MPC) uploaded all the transcripts into NVivo version 12 to facilitate data organisation and familiarisation. A thematic analysis approach was used to identify patterns and themes related to factors supporting and hindering integration of interactive playful parenting for ECD in community systems for health in Zambia. Thematic analysis approach was adopted because it facilitates identification, analysing, and reporting patterns or themes within data [18]. The first step involved reading of the transcripts, which enabled us to identify phrases and concepts within the data. These helped in developing codes which were grouped into subthemes and themes based on the similarities and differences. The developed thematic framework was revised based on discussions with co-authors. The developed themes were organised based on Atun’s Integration of Innovation into the Health System Framework. The coding process was performed based on the substantive coding structured. The analysis report was based on the thematic findings generated from the coding process.

### Findings

The findings are presented using Atun’s Integration of Innovations into the Health System conceptual framework, highlighting the factors shaping the integration of playful parenting to promote ECD in rural health system in Zambia (see Table 2).

**Table 2:** Conditions shaping levels of integration of ECD

Domains	Themes
Nature of the problem	• Recognition of ECD as cross cutting issue • The need for delivery of ECD interventions fostering playful interactive parenting • The need for trained manpower to deliver ECD
Attributes of the interventions	• Design of ECD Training Package • Providing capacity building ECD Training Distribution of ECD training materials and resources
Adoption systems	• Perspectives of policy implementers • Perspectives community leadership on ECD interventions • Perspectives parents on ECD activities • Perspectives teacher’s on ECD interventions
Broad system characteristics	• District-based multisectoral collaboration in delivery of ECD • Community-based collaboration, education, and linkages on ECDs • Availability community structures shaping delivery of ECD activities • Role of school administration in coordinating ECD • Role of healthcare workers in providing supportive supervision for delivery of ECD
Broad context	• Policy and legal environment • Political commitment to deliver ECD interventions • Inadequate allocation of human, financial resources, and materials • Lack of ECD infrastructure development

### Nature of the problem

Participants identified three key challenges affecting the integration of ECD interventions: limited recognition of ECD as a cross-cutting issue; the need for delivery of ECD interventions fostering playful interactive parenting; and the need for trained manpower to deliver ECD.

### The need for ECD programme

Participants emphasised that though ECD interventions are crucial for children’s social development and wellbeing but remain poorly integrated into the rural community systems for health. The multisectoral nature of ECD interventions made collaboration essential for successful implementation. A caregiver expressed the need for structured ECD programming:

“We always talk about how we are supposed to take care of our husbands, but we have never talked about how to take care of our children…We would like that programme to come. If you can bring it for us that can be better. We have a lot of mothers who have new-born babies here; so, if we educate each other, they can also go out there and educate other mothers.” (29 FGD, Care Givers).

The interactive playful parenting programme was recognised as requiring multiple stakeholders’ involvement. The participants supported the integration into the rural community systems for health as it was perceived to promote around development including nutritional, physical, physiological, and emotional development of children. This multisectoral recognition of ECD made it easier for different actors collaborate in integration of the interventions in rural community systems for health including health facility, ECD centres, communities and homes. The quote illustrates how ECD training helped parents prepare nutritious meals using locally available ingredients:

It (ECD) is really something that is very educative to our parents around this area in the sense. Through the same program they have learnt how to prepare very nutritious meals to feed the children even using locally available things like ground nuts etc. and they even prepare a balanced diet maybe cooking porridge for example using ground nuts they balance up things do that they feed the child on a very nutritious meal that will make a child grow holistically… physically and mentally health that is what these people have done (01, IDI, NHC Representative).

“And these things it’s a very good in the sense that they have even encouraged the multi-sectorial in bringing up children in health sector as a part, agriculture sector as a part, the teachers, the education as a part all of us come together to see how we can make this child grow to a marvel community he or she lives. So, we have really been assisted through this program.” (01, KII, School Representative).

### The need for trained staff to deliver ECD

Most actors involved in ECD implementation had inadequate capacity to deliver the interventions in different settings. The recognition of lack or inadequate knowledge in ECD including playful interactive parenting created impetus for developing the training programme for all human resources from different sectors including health, education, community development and agriculture. While staff capacity is limited, CBVs help fill the gap by responding quickly to community needs:

“Quite alright, we can go but its tedious. If you look at the district and how wide it is and we go and say, ok, we need to do this work maybe, maybe in a week. We’ll not do that but because of the CBVs we can just call them oh this is what we need. This is the information we need and maybe within a week or less than that” (04, KII, Health Sector Representative).

### Attributes of the intervention

This section highlights the attributes of the ECD interventions including such as design of ECD training package, providing capacity building ECD training Distribution of ECD training materials and resources in details.

### Design of ECD training package

When the formative need assessment was conducted, the ECD training curriculum for CBVs was designed. Respondents reported that the content included maternal, and child health, infection prevention, nutrition, growth monitoring and playful parenting techniques. Thus, the training programme focused on building capacity on the priority areas. These activities were emphasised during the training to prepare the CBVs to carry out these activities in the community health settings, clinic, community, homes, and schools. While the training covered key areas, participants emphasised its practical application in community settings: “I have been trained in ICCM, this malaria, malaria we are treating at home. Somebody comes at my place I treat him, or I test him, if found positive I give them medicine. Like every month we do growth monitoring, when we are doing growth monitoring, that’s where we have a huge (*15*,* IDI*, NHC Representative).

Participants appreciated the module’s practical design, especially maternity visits:

“ECD module. The module is based on four major concepts, communication, feeding or nutrition, prevention, and responding to illness. Start practical’s and practical we normally visit at maternity where we can see the neonate, those children that are just born and so on. Basically, then you go there we look at the position of the mother breastfeeding the baby” (09, KII, Policy Implementer).

Participants stated that they received knowledge and skills on educating the parents and caregivers on child development stages and how the community can help their children develop as expected.

“To teach parents with children to let them go back to school or for the playful parenting programme…” (24, FGD, CBVs).

### Providing capacity Building ECD training

The CBVs perceived the training as appropriate. Respondents reported that district-level managers participated in the CBV training that focused on family planning, nutrition, and on early childhood development. The training equipped them with competencies and knowledge that enabled them to cascade the programme to the community as reflected below. However, the training duration was short. Further, training opportunities on ECD were also missing. Some local trainers from district health office continued to support CBVs who needed more technical help to implement ECD in the local health systems. Despite gaps in ECD-specific training, CBVs appreciated the support and used it to engage their communities:).

“We are very much happy and grateful, we have learned a lot and now, we are trying to teach men in our communities that it is important for a man to play with a child at home, communicate with each other and build a relationship between a father, mother and a child…” (34,KII, NHC Representative).

### Distribution of ECD training materials and resources

Participants narrated that educational materials for CBVs working under the Ministry of Health were provided. Some of the IEC materials included picture and manual books (age-appropriate content and reporting forms). While CBVs have access to some instructional materials, participants noted that field-level implementation is often disrupted by lack of resources, as one participant explained:

“Manual books, yes, we use manual books. It has got stages of children, maybe 1, 2, 3, 4, and 5 years. So, we teach them according to the age of the child.” (34, IDI, NHC Representative).

This highlights a challenge in operationalising plans but also hope in the foundational tools already in place.

“No, it’s not like that we plan as such, but when it comes to the groundwork it’s different because we have no resources to move but we may plan to say quarterly let us do this ICCM supervision. For example, when global fund was supporting us, we were maintaining but, for example, this year we are not.” (09, KII, Government Representative).

### Adoption systems

We highlight the perspectives of different stakeholders, including community leadership, parents, and CBVs, influencing the integration of interactive ECD programs in rural community systems for health. These are discussed in detail below.

CBVs played a crucial role in identifying families of children with health such as nutritional problems. They provided counseling and education and helped parents rehabilitate their children through the provision of good nutrition. As one of the participants reported, CBVs were involved in identifying nutritional needs and linking families to primary health care. As one of the Participants emphasised that CBVs are instrumental in identifying and responding to child nutrition issues at the household level:

“They even go to teach other families which have children who are malnourished. They go to assist that family so that the child is assisted to grow physically and mentally health. That is what these people have done” (16, KII, Representative).

Learning through play is a critical aspect of early childhood development and education. Respondents indicated that the pupils that were exposed to ECD were more likely to participate better during the school activities and had also better learning outcomes or performance compared to children without the training. A school representative described how exposure to ECD positively influences children’s performance and engagement:

“This programme fits in a very good way kids who have gone to ECE and EC perform better than those who have not gone through this education… They do very well in school, they and active activities and zeal in whatever is going on around them” (16, KII, Representative).

### Perspectives community leadership on ECD interventions

The traditional leadership including chiefs and headmen, have the power to influence their subjects to participate and convince community to accept any intervention. The whole community come to listen to the authority and the mobilisation is usually acceptable because the leadership is trusted. A caregiver described how traditional leaders play a central role in mobilizing community support for ECD programmes:

“R2: When you see the chief and the headman come, the meeting will be successful because the headman is the one that see what happens in the village. The chief is just informed and encourages us to welcome the programme.” (18, FGD Caregivers).

Furthermore, the facilitators (CBVs) of ECD intervention must come from the same community. The CBVs coming outside the village of the parents were more likely to be rejected by the community. The local ones were preferred because they are familiar with the community networks, the dos, and don’ts and they are perceived as role models. The following quote illustrates how parents value familiarity and cultural alignment in ECD facilitators:

“R3: The person teaching should be someone from within not someone from far. Not like the programme is here and the person who’s supposed to teach is in Katete.” (18 FGD Caregiver).

### Perspectives parents on ECD activities

Good nutrition and feeding practices are one of the key pillars of early childhood development. The respondents confirmed that CBVs were involved in teaching parents from either home, play parks or health facilities on best feeding and nutritional practices that could enhance child health. While CBVs are actively promoting nutrition, some parents noted gaps in understanding and application, as one caregiver explained:

“R5: They (CBVs) use some books, which has some pictures they normally show us to say if you feed your child very well, this is how your child will look very healthy, but if you do not feed your child, this is how also the child will look when he or she is lacking food.” (26, FGD Caregivers).

Participants reported that ECD enhanced playful interactive opportunities between parents, caregivers, and fellow children to promote child growth. The programme also gave opportunities for children to interact with their friends at the play parks through various sports disciplines including football. This was appreciated because young children are difficult to take care of if there are less or no playful interactive activities. However, some participants noted inadequate play parks for children to play inhibited full participation of eligible children into the programme. The following quote illustrates how caregivers view play as essential to child growth and social development:

“R1: We were happy indeed to hear about this (programme). It’s one way a child can play with the parents and that way a child won’t be a troublemaker to the parents when they are being found at the play park. It helps children come together and play instead of making trouble. Behind my house, there is a playground where children have made a soccer team. They need a place where they can be playing and developing themselves in the long run, no parent restricts their own children;” (18, FGD Caregivers).

### Perspectives teacher’s (CBVs) on ECD interventions

Good nutrition was noted as a catalyst for children development and growth. Participants reported that the CBVs are playing a critical role in educating the community on ECD activities including promoting good nutrition. They help the parents through the ECD centres on how to prepare nutritious food children. Moreover, they were also seen educating parents on the best practices of feeding children. A school representative shared how CBVs are helping parents improve child nutrition through ECD centres:

“Is very educative to our parents around this area…through the same program they have learnt how to prepare very balanced diet and nutritious meals to feed the children even using locally available things like ground nuts…that will make a child grow holistically” (16, KII, Representative).

However, the parents also noted that some CBVs had limited knowledge on ECD. Thus, they recommended more training to enhance their capacity to deliver ECD lessons as expected. Participants highlighted gaps in CBVs’ understanding of ECD concepts and called for capacity-building:

“R5: They teach us, but they also need to be educated more so that when they come to teach us, they have full information, and they are about to deliver nicely” (26 FGD Caregivers). “To be honest, I think we are not because we haven’t covered some areas, unless teaching we are well-qualified” (37, IDI, NHC Representative).

### Health system characteristics

This section focuses on highlighting the district-based multisectoral collaboration in delivery of ECD, community-based collaboration, education, and linkages on ECDs, availability community structures shaping delivery of ECD activities, role of school administration in coordinating ECD and the role of healthcare workers in providing supportive supervision for delivery of ECD.

### District-based multisectoral collaboration in the delivery of ECD

Participants reported that the ECD has a district and community-based collaboration in the delivery of ECD-related activities. The district level committee consist of health, social welfare, nutrition, education, and private sectors to promote and oversee implementation.

District level committees were thought to be more active compared to committees formed at the community level. The district level structure supports the selection, training, and monitoring of ECD activities within the district. Participants highlighted the role of district-level committees in overseeing ECD implementation and volunteer selection:

“We have a multisectoral committee that helps to coordinate the activities of all these programmes that require input of various departments, to which we belong and then it comes to selecting which community-based volunteers should participate. Like for instance, the training for caring for care giver for child development. We are involved in also identifying from our end. Also, what we did first, we marked the volunteers to screen them from those that have multiple affiliation to say no, this is a CWAC is also a SMAG is also what.” (2, KII, District Programme Implementers).

### Community-based collaboration, education, and linkages on ECDs

Participants reported that CBVs conduct health education and outreach on ECD-related and child health-related activities. CBVs (MoH) were involved in registering eligible women into the program and discussing ECD-related topics from the manual. Additionally, the focus for outreach includes sensitisation to encourage the community to ensure that they observed water sanitation and hygiene, prevention of malaria and maternal and child health-related programmes. The following quote illustrates how CBVs actively engage communities in health promotion and ECD awareness:

“We go in the community and advice community members to sleep under a mosquito net, especially for pregnant mothers…we go in the community and advice community members to sleep under a mosquito net, especially for pregnant mothers” (30 FGD CBVs Prisons Facility).

CBVs creates a bridge between the community and health facilities. After conducting home visitation, they able to identify eligible caregiver, enrol them into the programme and children with health problems are linked to the health facilities for further management. Furthermore, CBVs also supervised water, sanitation and hygiene facilities, prevent infections and report complex issues to community leaders and health facilities to ensure that these are dealt with on time. Participants emphasised the crucial role CBVs play in linking households to health facilities and managing local health risks:

“We have already enrolled some women, I can say we do some appointments with them, we go and visit them and teach them through our manual books, what is important being with a child or anything concerning the child… We work as a link between the facility and the community. So, we look after maybe boreholes, wells, things concerning diseases maybe there is an outbreak in the community” (34, IDI, NHC Representative).

### Availability community structures shaping delivery of ECD activities

The existence of community structures opened for communities to air out their views regarding the implementation of the programmes. The community were able to report any challenge or concerns faced by the local health committees, chiefs, headmen who were able to speak on behalf of the community.

“For this programme to carry weight, we need to involve the chief from the start. Also, the headmen so that it carries weight. So, the chiefs and the headmen are needed for this programme to go forward” (11, IDI, Caregivers).

Coordination of the ECD intervention at the community level was essential to promote the smooth implementation avoid duplication and fragmentation of activities, and for CBVs and coordinators of ECD at the community held meetings to share experiences, challenges, and ways of solving them. However, community structures were sometimes weak, leading to instances where CBVs operated independently and developed their own schedules. A CBV representative described how various community structures are collaborating to support ECD:

“These institutions include, Ministry Education, and Health, churches, chiefs, CBVs, and a lot more, all these are working together to promote the programme. So, the groups are many that have come in, you come together (all groups). It helps to learn from one another and share more ideas on how to do things to achieve your goals. (13, CBVs Representative).

### Role of school administration in coordinating ECD

Supervising the CBVs implementing early childhood development in many centred was crucial to ensure adherence to the protocol. The zonal committee was established to supervise the work of volunteer teachers (CBVs) facilitating ECD lessons across centres. However, the standardised coordination mechanism was lacking, whereby all the CBVs and other community actors could come together and discuss issues affecting the implementation of ECD programmes. While zonal committees exist, participants noted the lack of a unified coordination structure, as one school representative explained:

“We have 10 zones, in this zone is one of the 3 pioneers for EDC and it coordinates the activities, and we have so many centres in our zone, and we are given a mandate to try to educate people on the importance of ECD and ECE so that we have sharp children. Here we coordinate all the activities, so, just around our school (16, Kll, School Representative).

### Role of healthcare workers in providing supportive supervision for delivery of ECD

Most CBVs meet their supervisors once a month during which the supervisor observes how they are delivering services, reviews their record keeping and provides on-site coaching and skills development. Despite having a hierarchy of supervision and workflow from the community to the district, the supervisory lines are not always linear, and supervision and reporting are not viewed as separate activities. The CBVs submit reports to the chairperson and consolidated at health facility into a single report every month. Participants described how healthcare workers supervise CBVs and receive reports:

“Our in-charge, yes even the reports that we prepare, we submit them to the in-charge. Yes, so that they see whether we are doing our job properly or not… a person who can or assigned to work in this village or community under malaria prevention when runs out of medicine, can refer and write a referral for that patient to go to the facility…” (06, FGD, CBVs Health Sector).

### Inadequate monitoring, and evaluation systems

The current monitoring meeting are inadequate to implement the programme. Thus, the current monitoring and evaluation was pragmatic have largely relied on pragmatic support from implementing partners. Novel innovations like ECD require a robust monitoring and evaluation system to track the progress of the intervention.

*“Implementing partners and Ministry of Health are supporting the programme because after the training*,* they have been monitoring. They had a meeting; they were just trying to follow up to see how far we have gone with the programme*” (33, IDI, Ward development committee Representative).

Another participant emphasised the need for more frequent engagement:

“Then conduct those major meetings like quarterly meetings. Like quarterly we can do quarterly those, but we feel visiting them every month will be ideal because of the component that they are doing especially under the issue of prayer and communication” (09, KII, District Programme Implementers).

### Broad context

This section presents the broad contextual factors including the policy and legal environment, political commitment to deliver ECD interventions, inadequate allocation of human, financial resources, and materials and lack of ECD infrastructure development delivery of ECD interventions.

### Policy and legal environment shaping ECD delivery

Early childhood development is essential in promoting the well-rounded development including physical, intellectual, social, affective, moral, and spiritual qualities of learners at pre-school level (aged 3–6 years) (Ministry of Education, 2013). However, many policies are silent on the delivery of ECD interventions to children under the age of 3 years in Zambia [19]. A life course approach has been acknowledged by social policy frameworks, such as the Integrated Framework for Basic Social Protection Programs, which can be seen as a chance for ECD initiatives like Playful Parenting to emerge [19]. However, the role of community-based [19] actors including parents involvement has not been mentioned though it is critical in preparing children for the next level, that is initiation into early childhood education [19]. This has a huge effect on the political and resources allocation to early childhood development in Zambia.

### Political commitment to deliver ECD interventions

The political will is crucial to support the development and maintenance of ECD centres. However, the participants reported that there is specific funding dedicated to support the development of ECD centres. Sometimes, implementing partners provide financial or material support to selected centres, but this support is often insufficient to meet all their needs. This is a community driven activity, which might be difficult to sustain. Although political will is appreciated, actual financial support remains a major constraint in the effective delivery of the ECD programme, as one key informant explained:

“The PTC, the responsibility is to run all the affairs of the Centre or the school. They look at the projects of the school, the development of the school when we talk of a community school, the government will not send funds to go and build that school. They will not send money for any structure there” (08, KII, District Policy Implementer).

### Inadequate allocation of human, financial resources, and materials

The current number of CBVs is not adequate, several CBVs are still complaining about heavy workloads and long distance to reach communities. The lack of funding has numerous ripple effects on the operations of ECD programmes including the distribution of training materials. Overall, CBVs in both districts felt that the materials and tools provided were not adequate to facilitate their work. The bicycle, medical supplies, and bags, and other materials. Another barrier affecting the delivery of ECD included CBVs lack of protective equipment rain boats and coats affect their work negatively, reports and books may get soaked, and sometimes, they do not work because they fear that they get soaked.

“We are many in one area others were forced to go look for clients elsewhere so that they manage the target they were given but then, transport was and is still the biggest issue. There are only 3 bicycles, and we have 39 CBVs so, transport is an issue. (22, FGDs, CBV Community Development).

Despite their dedication to community work, CBVs continue to face major resource constraints, as one participant noted:

“ECE centres and all activities are being carried out despite them not being sponsored or structured like some of the centres. They have been given materials they have even constructed some school structures for the activities that will enhance good growth” (16, KII, School Representative).

### Lack of ECD infrastructure development

The play parks to promote children’s playful interactive session exist but they are few. Some have damaged without being maintained. One caregiver expressed concern over the deterioration and need for expansion of these facilities:

“R3: At the school, there is a play park. It started well but now it’s gone down, were there are four or five villages they put a play park now those play parks are damaged” (18, FGD, Caregivers).

## Discussion

We employed Atun’s conceptual framework on the integration of innovation into the health system [15] to analyse conditions influencing the integration of interactive playful parenting for ECD and health in community systems for health in Zambia. This study identifies several challenges that limit the effective integration of ECD programmes at the community level. The main barriers include limited access to ECD interventions, a shortage of trained staff, inadequate infrastructure, and various community-specific challenges that affect programme implementation. Additionally, the study highlights that optimal implementation of ECD interventions requires strong collaboration among policy implementers, community leaders, parents, CBVs, and teachers.

Interactive playful parent-child engagement is critical but underutilised component of ECD. The Ministry of Health in collaboration with stakeholders developed and integrated the ECD intervention in selected health facilities within the health system to enhance playful parent-child interaction. The involvement of the CBVs in the delivery of ECD services facilitated increased coverage of the intervention. This engagement emerged as a solution in efficient delivery of the intervention in constraint resources including materials and inadequate healthcare providers. Their involvement also enabled targeted and timely interventions aligned with the needs and aspirations of local communities [20].

The integration of ECD into rural community systems for health requires trained healthcare providers, CBVs, the provision of training materials and resources to support its implementation. This finding is consistent with studies conducted in sub-Saharan Africa, which indicate that the successful integration of ECD interventions depends on supportive conditions including caregiver training, home and community-based services, and engagement of local stakeholders [21, 22]. In Ethiopia, for example, the integration of ECD included nutrition and psychosocial support, which improved child development and health outcomes [23]. Similarly, evidence shows that a lack of parental awareness of the benefits of the interventions hinder the active participation, resulting in limited of programme ownership and sustainability. This highlights the importance of community-based education and sensitisation efforts to enhance acceptability and uptake of services.

The study examined the adoption of ECD interventions by various stakeholders, including policy implementers, community leadership, parents, and teachers. Evidence from health innovations studies suggests that for integration to be optimal, the adopting system or context must be conducive in terms of skills, resources, supportive leadership, values, goals and regulations [24]. Our findings indicate that training of CBVs motivated them to deliver the interventions in local settings, schools, facilities, and communities. The recognition of ECD as a valuable intervention by policy implementers significantly shaped the level of support and acceptance of the programme [5]. The involvement of the community leadership, particularly chiefs and headmen, was critical in gaining community trust and ensuring intervention uptake. Their endorsement and support of ECD interventions, influenced community members to accept the intervention. Conversely, the lack of support and endorsement from key actors, can contribute to suboptimal integration, as interventions that are not prioritised by local decision-makers may struggle to gain traction, particularly in resource-limited settings [24].

The local health systems determinants also affected the extent to which ECD was integrated into the local settings. The role played by the district-level leadership in supporting the health facilities, schools and community-based structures, helped to strengthen relationships in the delivery of ECD. The district level structures played a crucial role in providing technical direction and support in ECD implementation. The CBVs worked in partnership with parents with support from community leadership to identify eligible households, provide health education and link those in need to either ECD centres or health facilities for the further management. Limited availability of healthcare providers hindered monitoring and supervision in the delivery of ECD services. This gap affected whether the programme was implemented as intended or not. Moreover, the CBVs could not receive the required help promptly to ensure optimal integration [25]. This largely due to lack of finances, which is currently heavily reliant on partner support.

The delivery of early childhood development (ECD) in local health systems is shaped by various broad contextual factors that have “influence its effectiveness. One such factor is the existing policies and laws related to ECD. In Zambia, while there is recognition of the importance of ECD for preschool-aged children, many policies are silent on interventions for children under the age of three years [26]. This lack of explicit attention to younger children and the role of community-based actors including parents, indicates a gap in policy implementation and coordination [26, 27]. Another factor influencing the delivery of ECD is the level of political commitment. Although political will is crucial for supporting the development and maintenance of ECD centres, the data show that there is inadequate specific funding dedicated to supporting these centres. In Ethiopia, Liberia, Pakistan, and Tanzania, because of the political commitment, the policymakers to respond to global efforts to integrate early childhood development into communities settings [28]. The weak mobilisation and collaboration with implementing partners such as NGOs, limited financial support towards the integration of interactive playful parenting due to limited ECD services [28].

The study also highlights the underfunding of ECD programmes, resulting in inadequate human, financial, resources, and materials resources, which hinders the delivery of ECD interventions. Insufficient numbers of community-based volunteers (CBVs) in Zambia result in heavy workloads and long distances to cover, impacting the effective implementation of ECD programmes [29]. Additionally, the lack of funding has ripple effects on the distribution of training materials and necessary tools for CBVs, hindering their ability to carry out their work effectively [25]. Furthermore, the lack of ECD infrastructure development poses a challenge to the delivery of ECD interventions. While there are play parks designed to promote interactive sessions for children, they are few in number and often poorly maintained [30]. This limits the availability of suitable spaces for children to engage in playful activities, which are essential for their holistic development [2]. Findings from Nepal and other resource-constrained settings confirm that inadequate investment in ECD infrastructure such as the provision of essential equipment like bicycles, medical supplies, and protective gear impacts child development outcomes [30].

### Limitations of the study

The study has certain limitations. As it focused on two selected districts, the findings may not fully represent the broader national context, limiting generalisability to other regions. The study was conducted within the context of a pilot project, meaning the experiences captured may differ from those in non-pilot ECD settings. Another limitation relates to potential response bias in qualitative interviews. Since interviews were conducted with programme implementers and other stakeholders, participants’ responses maybe influenced by their roles and responsibilities. This social desirability bias may have led participants to provide responses that aligned with programme expectations rather than offering an objective assessment of challenges faced. Additionally, some CBVs were actively engaged in multiple programmes under different ministries. This overlap may have blurred the boundaries between specific ECD interventions and other child health programmes, making it difficult to isolate the specific contributions and challenges related to the Playful Parenting ECD interventions. Despite these limitations, a key strength of this study is its inclusive engagement of diverse actors, including district officials, healthcare providers, CBVs, caregivers and community leaders. This approach facilitated an in-depth understanding of the conditions shaping the integration of interactive playful parenting in promoting ECD and child health. Future research should explore comparative studies across multiple districts, examining variations in ECD integration and the scalability of the intervention in different resource-constrained settings.

## Conclusions

This study sought to understand the conditions influencing the integration of interactive playful parenting for ECD and well-being in community systems for health in Zambia. The findings highlight several challenges, including, limited trained CBVs, inadequate distribution of resources and insufficient ECD infrastructure. To improve ECD service delivery, targeted interventions are required. These include expanding training opportunities for healthcare providers and CBVs; enhancing stakeholder collaboration among policy implementers, CBVs, community leaders, parents, and teachers; and strengthening healthcare managers’ supervision to support community-based implementation of ECD activities. Furthermore, increasing stakeholders’ collaboration policy implementers, CBVs, community leaders, parents, and teachers to enhance adoption and collective approach in the delivery of ECD in Community systems for health. This includes healthcare managers supportive supervision in promoting community-based implementation of ECD activities. The broad policy and contextual factors also play a critical role in shaping the optimal delivery of interactive playful parenting for ECD and health. Refining policies and laws through a bottom up consultative can enhance the delivery of ECD services. Additionally, engaging political leaders to advocate for increased resource allocation and infrastructure development will enhance inclusive ECD access. Further studies should investigate the role of multisectoral collaboration in enhancing delivery ECD services in resource-constrained settings.

## Supplementary Information

Supplementary Material 1.

## Data Availability

The study data can be requested from the corresponding author ([paul.malizgani@umu.se](mailto: paul.malizgani@umu.se)).

## Abbreviations

CBV: Community Based Volunteers
CHWs: Community Health Workers
CWAC: Community Welfare Assistance Committee
ECD: Early Childhood Development, ECE: Early Childhood Education
FGD: Focus Group Discussions
KII: Key Informant Interviews
LMIC: Low Middle Income Country
MOE: Ministry of Education
MOH: Ministry of Health
NGO: Non-Governmental Organisation
PTC: Parents and Teacher Committee
UNICEF: United Nations Children’s Emergency Fund
WHO: World Health Organisation
SMAGs: Safe Motherhood Action Groups
GMPs: Growth Monitor Promoters
MCDSS: Ministry of Community Development and Social Services
