# Supplementary material for: The role of community-based volunteers in integrating interactive playful parenting interventions for early childhood development in rural community systems for health in Zambia: a qualitative study
Source: BMC Prim Care. 2025 Dec 2;26:402. doi: 10.1186/s12875-025-03119-y (PMC12723914; doi:10.1186/s12875-025-03119-y)
Supplement: Supplementary file 1 — Supplementary Material 1. [file 12875_2025_3119_MOESM1_ESM.docx]

**Key Informant interview guide (Health facility in-charges, head teachers, and community welfare assistants)**

**Study: Mapping of Community Based Volunteers in Katete and Petauke** **under the LEGO Playful Parenting Programme**

Hello. My name is [*Researcher’s Name*] and I work with the University of Zambia-School of Public Health and UNICEF. I would like us to have a discussion on Community Based Volunteer programmes that are being implemented in Katete and Petauke. During the discussion, I will ask you questions on the range of tasks and duties covered by the different CBVs, training received, supervision and reporting procedures and the learning platforms available for CBVs.

Please be assured that your views are confidential and any information I collect will not be saved in your name so no one will know it was you. The information you provide will guide the implementation of early childhood development programmes that may be implemented in your district in the future. You can also stop the interview anytime without any consequences. We will record the discussion to ensure that we capture your views correctly. Our session will last for about last between 1-1.5 hours. Do I have your permission to record and start the discussion?

**Introduction.**

1. Please tell us a little bit about yourself and the role that you play in the implementation of Lego playful parenting programme within this district?

**Appropriateness and acceptability of the programme**

1. To what extent do you think that the activities covered under the programme are compatible with;
   1. The goals and objectives of your organization?
   2. Community based volunteers?
   3. Parents and care givers within your district?
2. To what degree would you describe the readiness of Community based volunteers to implement the playful parenting programme?
   1. Why would you say that this is the case?
   2. Are there specific aspects of the programme that were well received by the CBVs? How about those that were poorly received?
3. How do caregivers and community members in your district perceive the programme?
   1. Has this always been the case or has their reception of the programme changed over time?
   2. If their reception has been good, what are some of the factors that have contributed to this?
   3. If their reception has been poor, what are some of the factors that have contributed to this?
4. How would you describe the adoption of the programme among parents and care givers within your district?
   1. Has the rate of adoption changed over time since you began implementation? What kind of changes have occurred?
   2. What are the factors contributing to this?

**Barriers and facilitators to the implementation of the programme**

1. Do you think that the programme has had an impact on the development of children in your area? Why do you feel this is the case?
2. Generally speaking what do you think of the programme and how it has been implemented?
   1. Are there any successes that you have experienced that you would like to highlight?
   2. Are there any facilitators that enhanced the implementation of the programme?
   3. What are some of the challenges experienced in the implementation of the programme? Probe from intervention components, CBV related issues, organizational challenges?
3. As we come to the end of the interview is there any important information that you would like to state that I may have left out?

Thank you very much for your time.

**Mothers and Care Givers Focus Group Discussion Guide**

Hello. My name is [*Researcher’s Name*] and I work with the University of Zambia-School of Public Health and we are carrying out the study on behalf of UNICEF. I would like us to have a discussion on Early childhood development activities that are being implemented by Community Based Volunteer in Katete and Petauke. During the discussion, I will ask you questions on the range of tasks and duties covered by the different CBVs and whether the services that they provide are suitable for you and your children.

Please be assured that your views are confidential and any information I collect will not be saved in your name so no one will know it was you. The information you provide will guide the implementation of early childhood development programmes that may be implemented in your district in the future. You can also stop the interview anytime without any consequences. We will record the discussion to ensure that we capture your views correctly. Please be assured that this recording will not be shared with anyone outside the research team and the data collected will only be used for this study. Our session will last between 1-1.5 hours. Do I have your permission to record and start the discussion?

**Introduction.**

1. Are you aware of a programme that is being implemented in your community called the LEGO playful parenting programme?
   1. What do you know about the programme? Probe for who is responsible for implementing activities, what services are provided as part of the programme?
   2. How long has the programme been running within your community?
   3. How did you become aware of the programme?

**Appropriateness**

1. Do community based volunteers come to your home to deliver any early childhood development messages or interventions?
   1. What messages or interventions have they ever provided to you from your home?
2. Aside from coming to your homes, do you also go to the hubs?
   1. What do you think of the conditions at the hubs? Probe for adequacy of the materials. Space, resources etc.
   2. Are there community based volunteers present in the hubs who provide instruction in Early childhood development related issues? Is the information provided at the hub similar to what is provided during home visits?
3. Do you feel confident in the ability of CBVs to deliver accurate information on early childhood development?
   1. What are some of the reasons why you feel this way? Probe for age of the CBV, cultural beliefs, level of education etc
   2. Do you feel as though they are an effective means to deliver p
4. To what extent do you feel like the activities supported under the programme suitable for you and other care givers within your community?
5. Looking back at the activities that are being implemented as part of the programme, are there some activities that you like?
   1. Why did you like these activities?
   2. Are there any activities that you did not like? Why was this the case?
   3. Are there any aspects of the programme that you feel can be changed to make it a better match to you and your community?

**Acceptability**

1. When you first heard of the programme, did you enroll your children immediately or did you wait for some time?
   1. If you enrolled them what motivated you to do so?
   2. If you waited for some time, what convinced you to finally enroll your children on the programme?
2. Are there caregivers within the community who refuse to enroll their children for the programme? What are some of the reasons that they give for refusals?

**Barriers and facilitators to the implementation of the programme**

1. Do you think that the programme has had an impact on the development of children in your area? Why do you feel this is the case?
2. Generally speaking what do you think of the programme and how it has been implemented?
   1. Are there any successes that you have experienced that you would like to highlight?
   2. What are some of the challenges experienced?
3. As we come to the end of the interview is there any important information that you would like to state that I may have left out?

Thank you very much for your time.
